# Septic complications are on the rise and aseptic loosening has decreased in total joint arthroplasty: an updated complication based analysis using worldwide arthroplasty registers

**DOI:** 10.1007/s00402-024-05379-2

**Published:** 2024-05-25

**Authors:** Georg Hauer, Laura Rasic, Sebastian Klim, Lukas Leitner, Andreas Leithner, Patrick Sadoghi

**Affiliations:** 1 Department of Orthopaedics and Trauma, Medical University of Graz, Auenbruggerplatz 5, Graz, 8036 Austria; 2 Department of Orthopaedics and Trauma Surgery, Musculoskeletal University Center Munich (MUM), LMU University Hospital, Munich, Germany

**Keywords:** Arthroplasty, Register data, Complication, Total hip arthroplasty, Total knee arthroplasty, Revision

## Abstract

**Introduction:**

A decade ago, a comprehensive study was conducted to investigate the reasons for revision surgeries and their respective frequencies in cases of total hip arthroplasty (THA) and total knee arthroplasty (TKA) based on a complication-based analysis of joint replacement registries. The aim of the present study was to determine whether the causes and risks of their occurrence have changed over the last ten years and to present an updated analysis.

**Materials and methods:**

A systematic review of national arthroplasty registries from seven countries examined the causes and rates of revisions of THA and TKA. The study focused on a descriptive analysis that provided an updated overview without statistical significance values.

**Results:**

The most common causes for revisions of THA were aseptic loosening (35.1%), deep infection (18.2%), dislocation/instability (15.9%), and periprosthetic fractures (11.4%). The most common causes for revisions of TKA were deep infection (21.6%), aseptic loosening (18.3%), instability (14.1%), and pain (10.9%).

**Conclusion:**

The findings of this study revealed significant shifts in the underlying causes of revision surgeries in the last decade. Notably, septic complications emerged as the predominant reason for revision of primary TKA, while they also gained prominence as a cause of failure of THA. Although aseptic loosening remains the primary cause for re-operation of THA, the relative risk has decreased for both THA and TKA.

**Supplementary Information:**

The online version contains supplementary material available at 10.1007/s00402-024-05379-2.

## Introduction

Due to the increasing absolute number of total joint replacements (TJR), also the number of revision TJR is rising and predicted to continue to rise [7, 8]. As it is to be expected that predominately younger age groups will be affected by revision surgery, a thorough understanding of the reasons is necessary [9]. The need for a revision has severe consequences for the patient’s quality of life and leads to high health care costs [18].

A decade ago, it was reported that the reasons for revision surgery differ considerably between total hip arthroplasty (THA) and total knee arthroplasty (TKA) [23]. Aseptic loosening, deep infection and pain without other cause were the main reasons for TKA revision surgery, whereas THA revisions were performed mainly due to aseptic loosening, dislocation and deep infection. Although aseptic loosening was the number one cause in both procedures, the likelihood was much higher with THA (55.2% vs. 29.8%). In contrast, the risk for septic complications in TKA was almost twice as high compared to THA [23].

The purpose of the current study was to evaluate the causes of revision of THA and TKA in the last decade and to compare them with the above-mentioned published data in order to identify any developments. A complication-based analysis was performed in the case of revision surgery of THA and TKA using worldwide arthroplasty registers by summarizing the relative likelihood of different causes for revision surgery, and to describe differences between the arthroplasties.

The hypothesis of the study was that the reasons for revision and their relative proportion have changed over the last ten years. It was assumed that recent innovations in material properties and prostheses design would lead to a decrease of aseptic loosening. However, we expected an increase in revisions due to periprosthetic infection, as modern diagnostic techniques allow higher detection rates.

## Materials and methods

The same methodology was used as in the previous report 10 years ago. The first study included registry data up to the year 2009, while the current study also covers the period from 2010 to 2020. The previous data were pooled into the current report, as the registry data do not allow an exclusive focus on the period 2010–2020, as the relevant data from all years since the establishment of the respective registry are included in the respective annual report.

The authors performed a systematic review of published national arthroplasty registers. National arthroplasty register reports were scanned for data concerning revision causes of THA and TKA. The latest annual reports were taken from the EFORT (European Federation of National Associations of Orthopaedics and Traumatology) Website of the Network of Orthopaedic Registries of Europe (NORE) [19].

A thorough review of several registers was conducted using the EFORT Website for European Arthroplasty Registers [3]. We prioritized the most recent and biggest data sets of each country in order to update the results from the abovementioned publication. The registers had to provide clear and unambiguous information regarding the total number and reason of revisions for total hip arthroplasty (THA) and total knee arthroplasty (TKA). Additionally, we focused on national registry reports that documented at least 90% of the relevant arthroplasties or high-value register reports of Type A.1.1.1.1 [10, 12].

Registry reports were excluded that did not meet our quality criteria or that did not report the number of primary arthroplasties, and the number of revision surgeries. From our evaluation, we identified seven arthroplasty registers that fulfilled the aforementioned criteria. These registers include England/Wales/Northern Ireland, Norway, Finland, Sweden, Denmark, New Zealand, and Australia [1–3, 15, 17, 20, 25]. Two independent reviewers extracted the data and any discrepancies were resolved by consensus or with the assistance of a senior author. The extracted data were then entered into a predetermined data sheet. The entire process followed the guidelines of the PRISMA statement (Preferred Reporting Items for Systematic Reviews and Meta-Analyses), an established evidence-based guideline for systematic reviews published by the CONSORT group [14].

The included datasets consisted of the following parameters extracted: Country, period of the report, number of primary implantations, number of revision procedures, number of aseptic loosenings and/or osteolyses, number of dislocations, number of deep infections, number of periprosthetic fractures, number of technical errors or failures, number of implant fractures, wear or pain without other reason.

To facilitate the comparison of results, we categorized the questionnaires from various registers into broader groups. This approach allowed for a more effective analysis. For instance, when encountering the term “incorrect axis,” we treated it as a “technical error” for consistency. The term deep infection also included revisions for septic loosening. In numerous instances, there were multiple potential causes for revision, making it challenging to adequately address them solely by selecting the most suitable reason for revision.

### Statistical analysis

The present report focused on descriptive analysis of the results without significance values and we reported relative revision rates in relation to all revisions of the reported year. The revisions were categorized by reason for each registry, and the totals were summed to establish an overall count. This count per reason was then divided by the total number of revisions to ascertain the relative proportion. Additionally, proportions were calculated individually for each registry to enable comparisons across all included registers.

## Results

Using register data from multiple countries including England/Wales/Northern Ireland, Norway, Finland, Sweden, Denmark, New Zealand, and Australia, we successfully identified the factors leading to the need for revision surgeries in cases of TKA and THA. The data consisted of 2,124,821 primary cases and 197,624 (9.3%) cases of revised total hip arthroplasty (THA) as well as 2,296,308 primary cases and 121,022 (5.3%) cases of revised total knee arthroplasty (TKA).

**Fig. 1:**
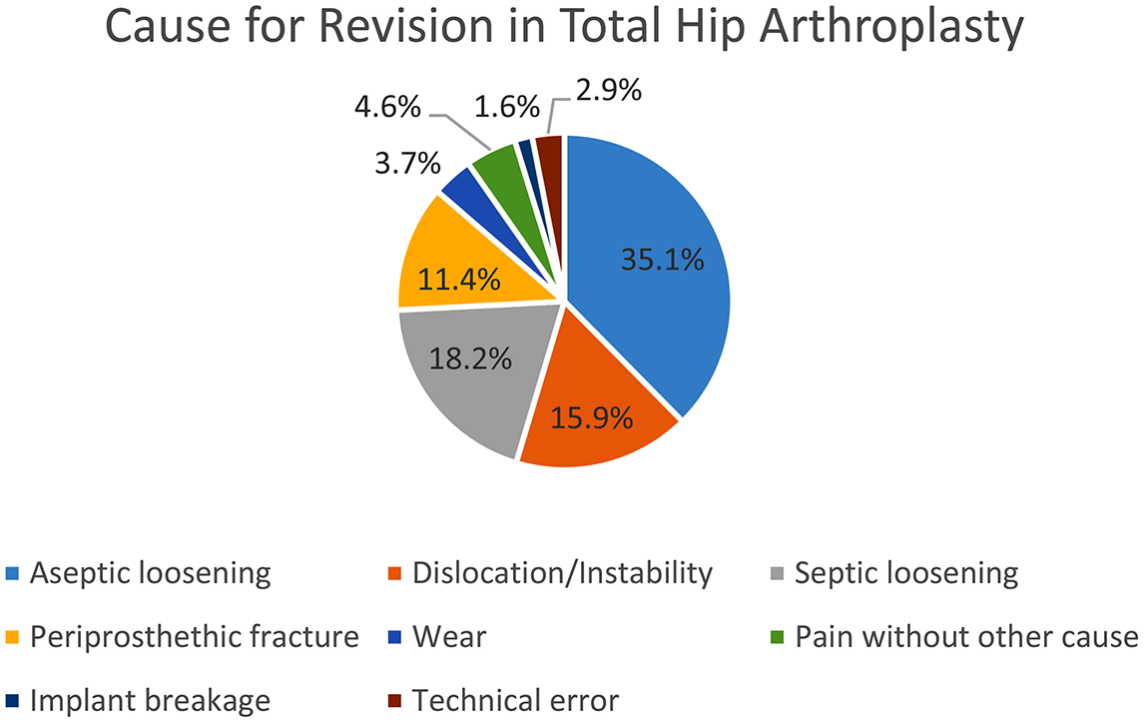
Diagram illustrating the causes for revision surgery after Total Hip Arthroplasty (THA) using worldwide arthroplasty registers in relative percentages with respect to the total number of revisions reported

**Fig. 2:**
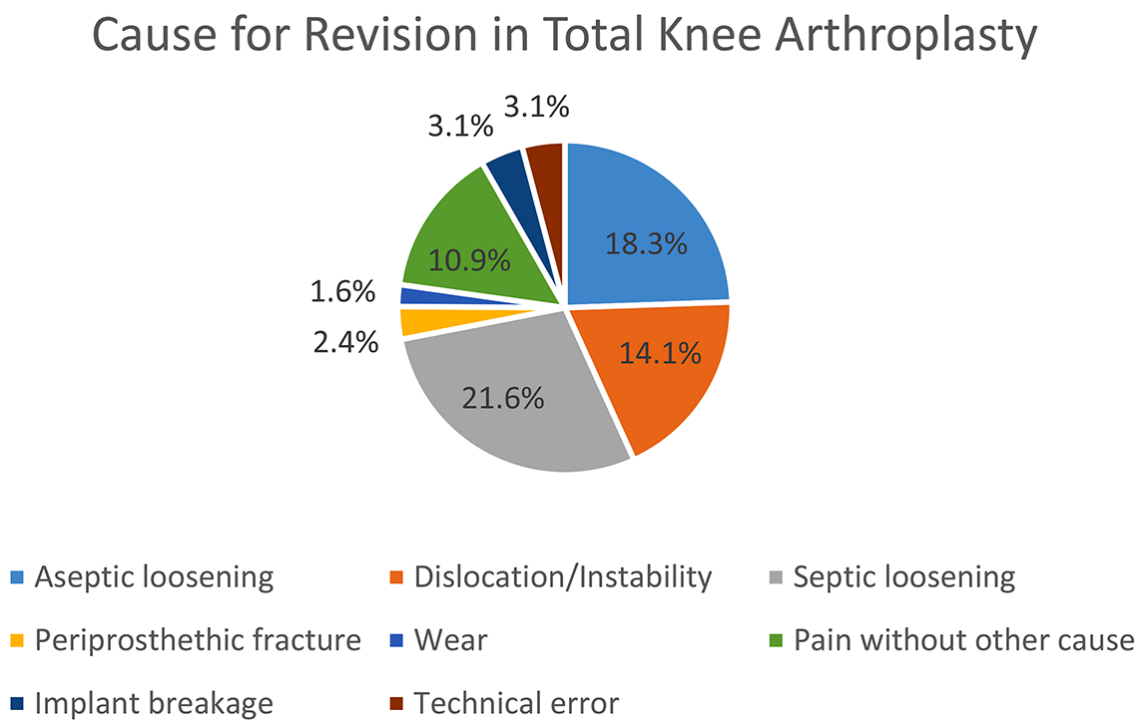
Diagram illustrating the causes for revision surgery after Total Knee Arthroplasty (TKA) using worldwide arthroplasty registers in relative percentages with respect to the total number of revisions reported

In THA cases, the most common causes for revision surgery, expressed in relative values, were aseptic loosening (35.1%), followed by deep infection (18.2%), dislocation/instability (15.9%), periprosthetic fractures (11.4%), pain without other reason (4.6%), wear (3.7%), technical error (2.9%) and implant breakage (1.6%). In TKA cases, the primary reasons for revision surgery, in relative values, were deep infection (21.6%), aseptic loosening (18.3%), dislocation/instability (14.1%), pain without other reason (10.9%), implant breakage (3.1%), technical error (3.1%), periprosthetic fracture (2.4%), and wear (1.6%). This information is further illustrated in Table 1; Figs. 1 and 2.

**Table 1:** Reasons for revision surgery after Total Knee Arthroplasty (TKA), Total Hip Arthroplasty (THA) using worldwide arthroplasty registers

Cause for revision	Total hip arthroplasty	Total knee arthroplasty
Aseptic loosening	35,1%^a^	18,3%
Deep infection	18,2%	21,6%
Dislocation/Instability	15,9%	14,1%
Periprosthethic fracture	11,4%	2,4%
Wear	3,7%	1,6%
Pain without other cause	4,6%	10,9%
Implant breakage	1,6%	3,1%
Technical error	2,9%	3,1%

^a^ Values represent percentage of cause of revision with respect to the total number of revision surgeries

In THA cases, aseptic loosening was the main reason in Norway (42.3%), England/Wales/Northern Ireland (41.7%), New Zealand (34.1%) and Denmark (21.5%). Deep infection was the main reason in Finland (27.8%), and had high proportions in Norway (23.2%), Australia (22.3%), New Zealand (18.8%) and Denmark (16.7%), however deep infection accounted for only 5.0% in England/Wales/Northern Ireland. Dislocation/instability was the main reason in Australia (22.5%) and was a dominant factor in the registries of Finland (26.5%), England/Wales/Northern Ireland (23.9%), Norway (19.2%) and Denmark (19.0%). Periprosthetic fractures showed an increase in every included registry compared to a decade before, with the highest proportion observed in England/Wales/Northern Ireland (25.7%).

In TKA cases, aseptic loosening was the main reason in England/Wales/Northern Ireland (44.6%) and Norway (17.9%). Deep infection was the leading cause in Australia (26.4%) and Finland (34.9%). Dislocation/instability was the main reason in Denmark (23.0%) and a main factor in Finland (32.2%), England/Wales/Northern Ireland (23.1%) and Norway (18.0%). Periprosthetic fractures, again, displayed an upward trend, although not as prominently as seen in hip prostheses, with England/Wales/Northern Ireland reporting the highest prevalence (10.0%).

## Discussion

The current study could demonstrate that there were notable changes in the underlying causes leading to revisions over the past ten years. The most important finding was that septic complications are now the dominant mechanism of revision surgery after primary TKA and are an increasingly important cause of failure in THA. Aseptic loosening remains the leading cause for re-operation after THA, however, the relative risk has declined for THA and TKA.

Compared to the data from the previous systematic review, the revision rate in the current data for THA decreased from 15 to 9%, and for TKA from 9 to 5%. For both knee and hip replacements, the relative risk of septic complications has increased compared to the data from 10 years ago [23]. Despite the widespread use of perioperative prophylactic antibiotics and other infection control measures in TJR, infections remain one of the most common major complications. In the study results from a decade ago, septic events accounted for 14.8% of knee replacement revisions, while now they have increased to 21.6%. This means that septic complications have become the most common reason for re-operation after TKA. A similar trend can be observed for hip replacements, where the percentage has increased from 7.5 to 18.2%. Aseptic loosening is still the most common cause for revision after THA, but its percentage has decreased from 55.2 to 35.1%. In knee prostheses, aseptic loosening has now become the second most common reason, accounting for a percentage of 18.3% compared to 29.8% ten years ago.

Consistent with the updated registry data presented here, a study combining local hospital data with national joint registry data also demonstrated that septic events were the most common cause of revision surgeries following TKA [6]. The authors complained that registries often under-report revisions particularly for periprosthetic joint infections (PJI). The reason behind this could be that septic revision procedures without the exchange of components are not recorded as reoperations in the register. In addition, emergency septic procedures often take place outside regular working hours, which can lead to missing entries in the register databases [27]. Efforts to improve the capture rate of PJI should focus on well documented reporting forms and a standardisation of the term revision operation across registries.

In another report using registry data from the National Joint Registry (NJR), Sabah et al. could demonstrate that aseptic loosening (20.7%) was the most common cause of revision knee replacement between 2006 and 2019, followed by infection (16%) [22]. However, cases of aseptic loosening have decreased in recent years, while infection rates have increased. In 2019, infection overtook other factors as the main cause of revision knee surgery. Lewis and colleagues reported similar trends as they saw rises in the proportion of revisions for infection and declines in the proportion of revisions for wear across the three registries they looked at [11].

Furthermore, registries typically have no defined criteria for PJI [16] which can contribute to the underrecognition of septic causes. Uniform and well-established criteria such as the Musculoskeletal Infection Society (MSIS) [21] and European Bone and Joint Infection Society (EBJIS) criteria [13], combined with improved detection capabilities, are necessary to detect PJIs that would have been classified as aseptic complications 10–15 years ago.

Aseptic loosening remains a dominant failure mechanism after TJR [5, 24, 26]. However, as we had assumed, there was a substantial decrease in the risk. Newer fixation techniques with enhanced osseointegration of cementless components, decreased wear rates, improved prostheses designs have contributed to lower aseptic loosening rates [4]. An alternative explanation could also be that aseptic loosening was often given as a reason for revision when it was actually due to another aseptic failure mechanism or because the primary implant was never secure. Aseptic loosening as a “catch-all” diagnosis may decrease as more detailed investigations are conducted and subcategories of aseptic causes of revision are explored in more detail [26]. Additionally, registry reports include revisions of implants that were implanted over 20 to 30 years ago. While this approach covers revisions of older implants and highlights failure mechanisms such as aseptic loosening, which are more commonly observed in long-term follow-up, it may not fully capture the failure mechanisms seen in newer prostheses [24].

In both our current and previous research, instability and dislocation were found to be a consistent factor contributing to failure. Surprisingly, an increase was observed in both THA and TKA. Instability and dislocation used to account for 11.8% of hip replacement revisions, while now they have increased to 15.9%. In TKA, the rate has increased from 6.2 to 14.1%. Significant efforts must be made to reduce preventable revisions, such as primary instability and malalignment, in order to lower the failure rate in this regard.

It is noticeable that the distribution of frequencies varies regionally. While the exact reasons cannot be determined solely from the registries, speculative hypotheses can be formulated. The registry of England/Wales/Northern Ireland for instance has very low rates of deep infection compared to the other registries. It could be that the capture rate of PJI is lower or that the data entry may not be entirely accurate. On the other hand, there are higher proportions of periprosthetic fractures in England. This could possibly be due to a higher frequency of prosthesis replacements in that region, whereas in other countries, alternative fracture treatments may be preferred, leading to the retention of the prosthesis. In such cases, entry into a prosthesis registry may not occur.

his study has some limitations. Based on the available data, it was not possible to differentiate between early and late reasons for revision. Additionally, it should be noted that the primary data in the arthroplasty registries were entered based on the subjective assessment of the revision surgeons and that the reliability and completeness of the information in the individual arthroplasty registries significantly affect the quality of this analysis. Thirdly, we did not focus on revision rates but only on revision cause. Fourthly, the most recent register data always include historical data since the foundation of each respective registry. Regarding reasons for revisions, it is not possible to exclude the last ten years separately. It is possible that documented septic complications have increased even more in the last ten years than we could demonstrate here. Fifthly, we also want to make clear that the registries are always evolving, and that data collecting and interpretation are constantly getting better. Sixthly, medical data is missing, and due to the heterogeneity of registry data, we cannot demonstrate how age, gender, body mass index, and comorbidities influence the reasons for revisions. Despite these limitations, the present study offers valuable and novel insights into the revision causes after TJR utilizing the largest dataset examined to date.

## Conclusion

In the field of arthroplasty, the reasons for revision have changed considerably over the last ten years. This study underlines the changing distribution of reasons for revision and highlights the need for continuous investigation and improvement. Continuous research is essential to adapt procedures and improve the outcomes of arthroplasty revisions.

## Electronic supplementary material

Below is the link to the electronic supplementary material.

Supplementary Material 1

Supplementary Material 2
